# Quantitative Changes in the Sleep EEG at Moderate Altitude (1630 m and 2590 m)

**DOI:** 10.1371/journal.pone.0076945

**Published:** 2013-10-22

**Authors:** Katrin Stadelmann, Tsogyal D. Latshang, Christian M. Lo Cascio, Noemi Tesler, Anne-Christin Stoewhas, Malcolm Kohler, Konrad E. Bloch, Reto Huber, Peter Achermann

**Affiliations:** 1 Institute of Pharmacology and Toxicology, University of Zurich, Zurich, Switzerland; 2 Zurich Center for Integrative Human Physiology (ZIHP), University of Zurich, Zurich, Switzerland; 3 Pulmonary Division, University Hospital Zurich, Zurich, Switzerland; 4 University Children's Hospital Zurich, Zurich, Switzerland; Simon Fraser University, Canada

## Abstract

**Background:**

Previous studies have observed an altitude-dependent increase in central apneas and a shift towards lighter sleep at altitudes >4000 m. Whether altitude-dependent changes in the sleep EEG are also prevalent at moderate altitudes of 1600 m and 2600 m remains largely unknown. Furthermore, the relationship between sleep EEG variables and central apneas and oxygen saturation are of great interest to understand the impact of hypoxia at moderate altitude on sleep.

**Methods:**

Fourty-four healthy men (mean age 25.0±5.5 years) underwent polysomnographic recordings during a baseline night at 490 m and four consecutive nights at 1630 m and 2590 m (two nights each) in a randomized cross-over design.

**Results:**

Comparison of sleep EEG power density spectra of frontal (F3A2) and central (C3A2) derivations at altitudes compared to baseline revealed that slow-wave activity (SWA, 0.8–4.6 Hz) in non-REM sleep was reduced in an altitude-dependent manner (∼4% at 1630 m and 15% at 2590 m), while theta activity (4.6–8 Hz) was reduced only at the highest altitude (10% at 2590 m). In addition, spindle peak height and frequency showed a modest increase in the second night at 2590 m. SWA and theta activity were also reduced in REM sleep. Correlations between spectral power and central apnea/hypopnea index (AHI), oxygen desaturation index (ODI), and oxygen saturation revealed that distinct frequency bands were correlated with oxygen saturation (6.4–8 Hz and 13–14.4 Hz) and breathing variables (AHI, ODI; 0.8–4.6 Hz).

**Conclusions:**

The correlation between SWA and AHI/ODI suggests that respiratory disturbances contribute to the reduction in SWA at altitude. Since SWA is a marker of sleep homeostasis, this might be indicative of an inability to efficiently dissipate sleep pressure.

## Introduction

Sleep at altitude is generally characterized by a shift towards lighter sleep, and diminished objective and subjective sleep quality [1]. Several studies have reported a reduction in the duration of slow-wave sleep (SWS) and rapid-eye movement (REM) sleep, an increase in the number of awakenings and arousals, and minutes of stage 2 sleep [2]–[6]. Because central apneas and periodic breathing increase at altitude, the sleep fragmentation observed at altitude may in part be due to arousals resulting from these events. A large field study at 4559 m found, however, that the number of arousals in the first nights at altitude was increased, although not in proportion to the increase in central apneas/hypopneas [6]. Moreover, oxygen saturation and not the apnea/hypopnea index (AHI) revealed a significant effect on slow-wave sleep. Therefore, it remains unclear whether breathing disturbances are the main trigger for reduced sleep quality at altitude, despite the observation that the number of apneas and arousals are altitude-dependent, worsening with increasing altitude. Moreover, published studies do not differentiate between the effects of altitude, other environmental factors (e.g., activity level) and acclimatization [5], [7].

One further limitation of the above-mentioned studies is that sleep at altitude has primarily been investigated using visually defined sleep stages which are dependent on characteristic features in the EEG, EOG and EMG signals. It is known that severe hypobaric hypoxia at high altitudes (>3000 m) results in evident changes in sleep architecture [2]–[5] and slight changes were observed at moderate altitude [8], [9]. More subtle, but physiologically relevant changes in the sleep EEG may not necessarily be detectable by sleep stage analysis.

One method to extract information from the EEG is power spectral analysis [10], [11], a quantitative summary of EEG measured cortical activity. An advantage of power spectral analysis is that it allows detailed investigation of the oscillations present in the EEG by decomposing the signal into its constituent frequency components. Thus, power spectral analysis provides valuable information that is lacking in sleep stage scoring and is more sensitive to subtle changes in sleep physiology.

Therefore, our aim was to examine the impact of altitude on sleep using power spectral analysis in a field study with a large sample size (n = 44) and a randomized cross-over design. Despite the popularity of recreational sports and stays at moderate altitudes (<3000 m), few studies have examined whether sleep is altered at moderate altitudes. We therefore compare sleep at a low altitude (490 m) with sleep at two moderate altitudes (1630 and 2590 m). Altitude dependent changes in sleep architecture, respiration and performance [12] as well as on vascular function, metabolism and systemic inflammation [13] were reported recently.

Our design allows us to take advantage of spectral analysis to uncover subtle but relevant changes to sleep in an altitude-dependent manner. Since sufficient, undisrupted sleep is essential for well-being and good motor and cognitive performance [14], altitude dependent sleep EEG alterations are of great interest. In addition, we examined the relationship between sleep EEG spectral characteristics and respiratory disturbances and oxygen saturation by correlation analysis. This allows us to investigate which physiologically relevant frequency bands are affected by these variables and in which direction. Our results revealed alterations in the sleep EEG at moderate altitude associated with respiratory disturbances and oxygen saturation.

## Methods

### Subjects

Fifty-one healthy men, living below 600 m, non-smokers, free of sleep complaints and medication participated in the study. Recruitment mainly consisted of an invitation via email, which was sent to students at the ETH Zurich and the University of Zurich. In addition, subjects were recruited using flyers. For further details on participant characteristics see Latshang et al. [12]. Because the focus of the present analysis was on spectral changes in the sleep EEG the exclusion criteria was a sleep efficiency <80% during sleep at 490 m and the first night at higher altitude (see below for study protocol) to assure sufficient data for the calculation of spectra. Based on these criteria seven participants were excluded (three at 490 m; two at 1630 m; two at 2590 m) resulting in a final sample size of forty-four men [mean age ± SD: 25.0±5.5 years] for the current analysis. Sojourns to altitudes above 1500 m during the last 2 weeks before the study period were not allowed. In addition, subjects had to abstain from caffeine and alcohol consumption and adhere to regular bedtimes (7 h, from 11 p.m. to 6 a.m.) for at least the 3 days/nights preceding study nights. During the study periods subjects were instructed to remain at current altitude and avoid extensive physical exercise. Compliance was tracked with an actimeter with a built-in altimeter and sleep logs. The study protocol was approved by the cantonal ethical committee, and participants gave their written informed consent and were monetary compensated upon completion of the study. The study was registered (clinicaltrials.gov; ID#NCT01130948).

### Study procedure

The study was carried out in the sleep laboratory of the Pulmonary Division of the University Hospital Zurich (Baseline, 490 m), at the Hochgebirgsklinik Davos-Wolfgang (1630 m), and at the mountain hostel Davos-Jakobshorn (2590 m), Switzerland. Subjects slept in individual bedrooms and light exposure was controlled at all locations. Time in bed during the study nights was 7 hours. Lights were turned off at 11 p.m. and on at 6 a.m.. The reason for limiting sleep to 7 hours was the circumstantial tests and examinations in the morning and afternoon (see [12], [13]).The protocol consisted of 5 study nights: one baseline night (490 m) and four consecutive nights at moderate altitude (two nights at 1630 m [N1, N2] and two nights 2590 m [N1, N2]) completed in a randomized cross-over design (Figure S1). Participants were divided into four groups based on the order of altitude exposure (group 1 (*n* = 11): 490 m - 1630 m - 2590 m, group 2 (*n* = 11): 490 m - 2590 m - 1630 m, group 3 (*n* = 12): 1630 m - 2590 m - 490 m and group 4 (*n* = 10): 2590 m - 1630 m - 490 m; Figure S1). Every evening before and every morning after a recording, participants completed comprehensive test blocks comprised of medical and cognitive examinations [12], [13].

### Polysomnographic recordings

During the seven hours of nighttime sleep, EEG (derivations C3A2, C4A1, F3A2 and F4A1, electrode positions according to the 10–20 system), submental EMG and EOG were recorded (Alice5, Philips Respironics AG, Zofingen, Switzerland). In addition, respiratory signals consisting of calibrated inductance plethysmography, nasal pressure swings and pulse oximetry [15]. The EEG data were sampled at 200 Hz (high-pass filter: 0.32 Hz; low-pass filter: 100 Hz; notch filter: 50 Hz).

Sleep stages (30-s epochs) and arousals were visually scored according to standardized criteria [16], [17].

### Sleep cycle analysis

Non-REM-REM sleep cycles were defined according to the criteria of Feinberg and Floyd [18]. A sleep cycle consists of a non-REM sleep episode (at least 15 min in duration) and the subsequent REM sleep episode (at least 5 min in duration, with the exception of the first REM episode). The last sleep cycle was considered to be completed if REM sleep was followed by at least 5 min of non-REM sleep. We corrected for skipped REM sleep episodes using the criteria of Carskadon and Jenni [19]. Because the majority of nights were comprised of at least three sleep cycles, we performed statistical analysis on the first three cycles (Table 1).

**Table 1:** Sleep cycle analysis.

		490 m	1630 m N1	1630 m N2	2590 m N1	2590 m N2	p^A^
**Number of cycles**		3.6 (0.06)	3.6 (0.06)	3.7 (0.05)	3.8 (0.07)*	3.9 (0.06)*	Altitude, Night
**Length of cycles (min)**	c1	88.9 (5.9)	77.2 (3.9)	82.5 (4.0)	82.4 (4.4)	82.1 (3.4)	ns
	c2	112.4 (5.0)	119.3 (5.2)	108.7 (4.1)	114.3 (4.4)‡	104.4 (3.3)‡	Night
	c3	109.0 (3.9)	114.7 (4.5)	106.9 (3.7)	108.1 (3.9)	110.4 (4.1)	ns
**Length non-REM sleep episode (min)**	c1	75.5 (4.6)	67.8 (3.4)	64.5 (3.5)*	68.3 (4.1)	66.6 (2.8)	Altitude
	c2	85.8 (3.8)	91.2 (4.1)‡	81.3 (3.0)‡	88.8 (3.7)‡	79.9 (2.5)‡	Night
	c3	84.5 (3.2)	80.7 (2.4)	73.9 (2.8)	76.7 (3.2)	77.5 (2.4)	ns
**Length REM sleep episode (min)**	c1	16.5 (1.9)	15.6 (1.3)	18.4 (1.7)	15.7 (1.5)	17.0 (1.6)	ns
	c2	25.8 (2.0)	28.8 (2.1)	27.3 (2.1)	24.9 (1.8)	25.1 (1.8)	ns
	c3	24.5 (1.7)	34.0 (3.0)*	33.0 (2.5)*	31.4 (3.9)	30.2 (2.0)	Altitude
**S2 sleep (min)**	c1	29.7 (3.2)	23.5 (1.9)	20.1 (1.5)*	25.5 (2.3)	22.3 (1.7)*	Altitude
	c2	43.2 (2.9)	47.0 (2.1)* ‡	37.9 (2.7)‡	53.3 (2.8)	46.6 (2.9)	Altitude, Night
	c3	53.6 (2.5)	49.4 (2.3)	51.5 (2.7)	49.5 (2.5)	49.8 (2.5)	Order
**SWS (min)**	c1	40.4 (2.4)	36.3 (2.3)	40.4 (3.4)	37.4 (2.5)	40.8 (2.0)	Night, Order
	c2	31.1 (2.5)	28.6 (2.2)‡	38.8 (2.2)* ‡	24.6 (2.0)*	25.5 (2.1)	Altitude, Night
	c3	17.3 (2.0)	18.6 (2.1)	15.2 (1.7)	15.7 (1.6)	17.0 (2.1)	ns

Data are provided as means (SEM; n = 44). *Post hoc* paired t-tests were performed if factor *Altitude* or *Night* (p^A^: significant factor is indicated; ns: not significant) of the mixed model ANOVA with factors *Altitude*, *Night*, *Order* or their interactions was significant.

* p<0.05 (paired t-test) altitude compared to baseline.

‡p<0.05 (paired t-test) first compared to second night at respective altitude.

c1–3: Sleep cycle 1–3; SWS: Slow wave sleep (stages 3 and 4); N1, N2: night 1 and 2 at higher altitude.

### Breathing variables and oxygen saturation

Analysis was performed as previously described [15]. A reduction in breathing amplitude to <50% of the preceding two minutes lasting longer than 10 s was defined as apnoea/hypopnoea. If the apnoeas/hypopnoeas occurred as a part of a periodic breathing pattern (at least three consecutive cycles), apnoeas/hypopneas of shorter duration (5–10 s) were also scored. The apnoea/hypopnoea index (AHI) and the oxygen desaturation index (>3% drop; ODI) were computed as the number of events per hour of sleep [15].

### Power spectral analysis

To perform spectral analysis with Matlab (The MathWorks, Inc., Natick, MA, USA) the EEG data were first exported from the Alice sleep software system to the European data format (EDF). EEG power density spectra of consecutive 30-s epochs (FFTW approach, Hanning window, averages of six 5-s epochs; frequency resolution 0.2 Hz) were computed. The three lowest frequency bins (0.2–0.6 Hz) were excluded from analysis because of their sensitivity to low frequency artifacts. Spectral data were analyzed up to 25 Hz. EEG artifacts were removed semi-automatically. Epochs were excluded whenever power in the 20–40 Hz and 0.8–4.6 Hz band exceeded a threshold based on a moving average determined over twenty 30-s epochs. Statistical analysis was performed on average power density spectra, calculated over the minimal common number of epochs of non-REM or REM sleep across all five nights of a subject. Spectra of the left and right hemisphere did not differ. Therefore, further analysis was restricted to left frontal (F3A2) and central (C3A2) derivations.

### Statistical analysis

**Figure 1:**
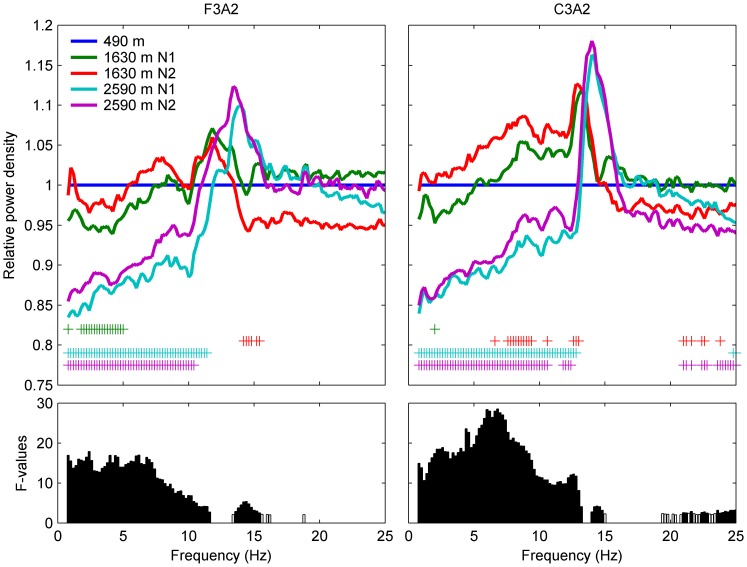
Relative non-REM sleep EEG power density spectra at moderate altitude. **Upper panels:** Spectra at altitude (1630 m and 2590 m, N1 [first night] and N2 [second night]) are plotted relative to baseline sleep (490 m; line at 1). Significant differences (p<0.05, post-hoc paired t-test) between baseline and altitude are indicated by “+” (n = 44). Frequency resolution: 0.2 Hz. **Lower panels:** F-values of the frequency bins with significant p-values for factor *Condition* (490 m N1, 1630 m N1, 1630 m N2, 2590 m N1 and 2590 m N2) of mixed model ANOVA with factors *Condition* and *Order*. F3A2: frontal derivation; C3A2: central derivation. Due to technical problems during N2 at 2590 m, the EEG data of two subjects were incomplete and these two nights were excluded from the analysis.

**Figure 2:**
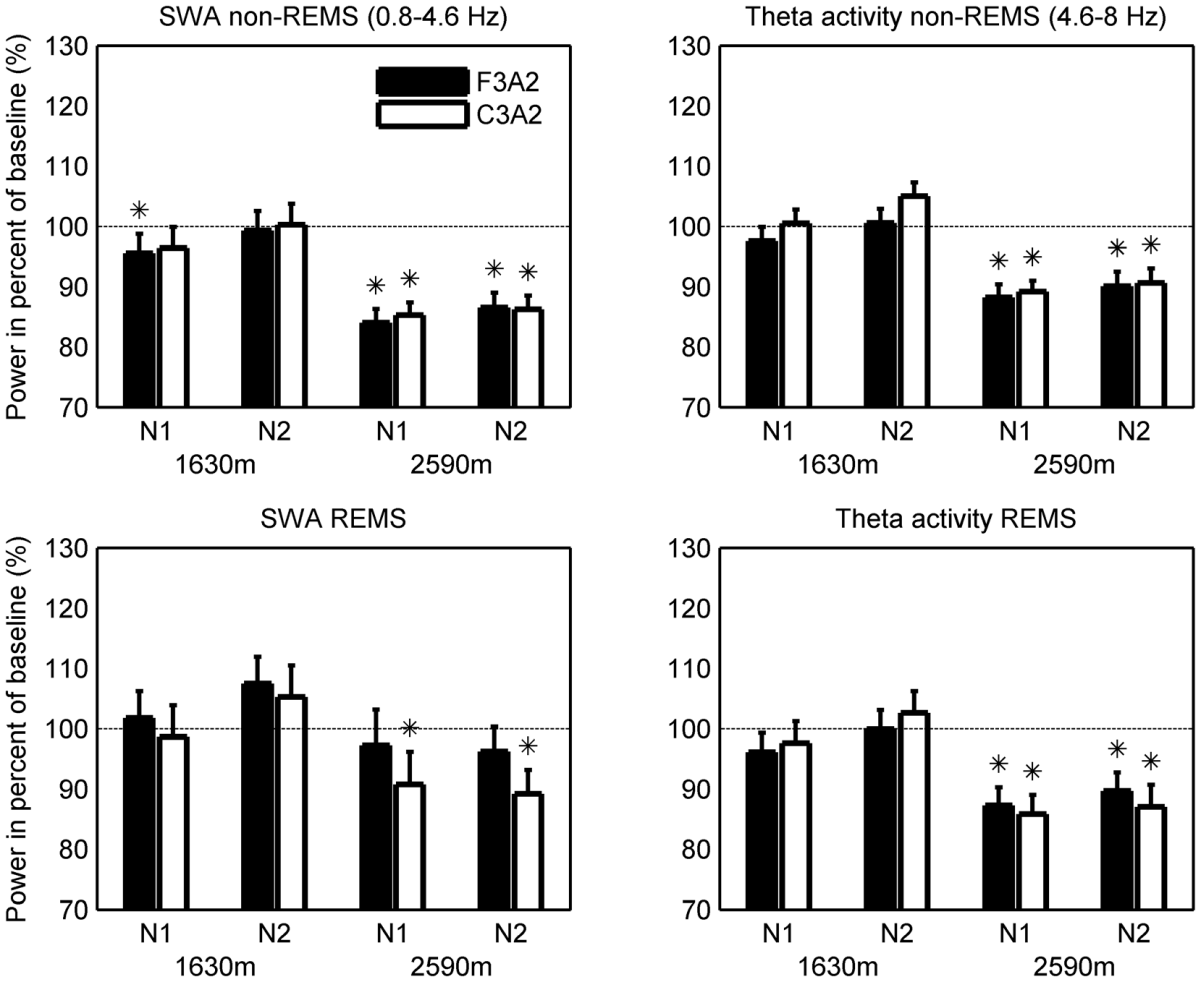
Relative slow-wave and theta activity at moderate altitude. Mean (SEM; n = 44) slow-wave (SWA; 0.8–4.6 Hz) and theta activity (4.6–8 Hz) in non-REM and REM sleep (REMS) at altitude (1630 m and 2590 m, N1 [first night] and N2 [second night] at a particular altitude) expressed as percentage of baseline sleep at 490 m (100%; dotted line). F3A2: Frontal derivation; C3A2: Central derivation. *p<0.05 (paired t-test altitude compared to baseline).

**Figure 3:**
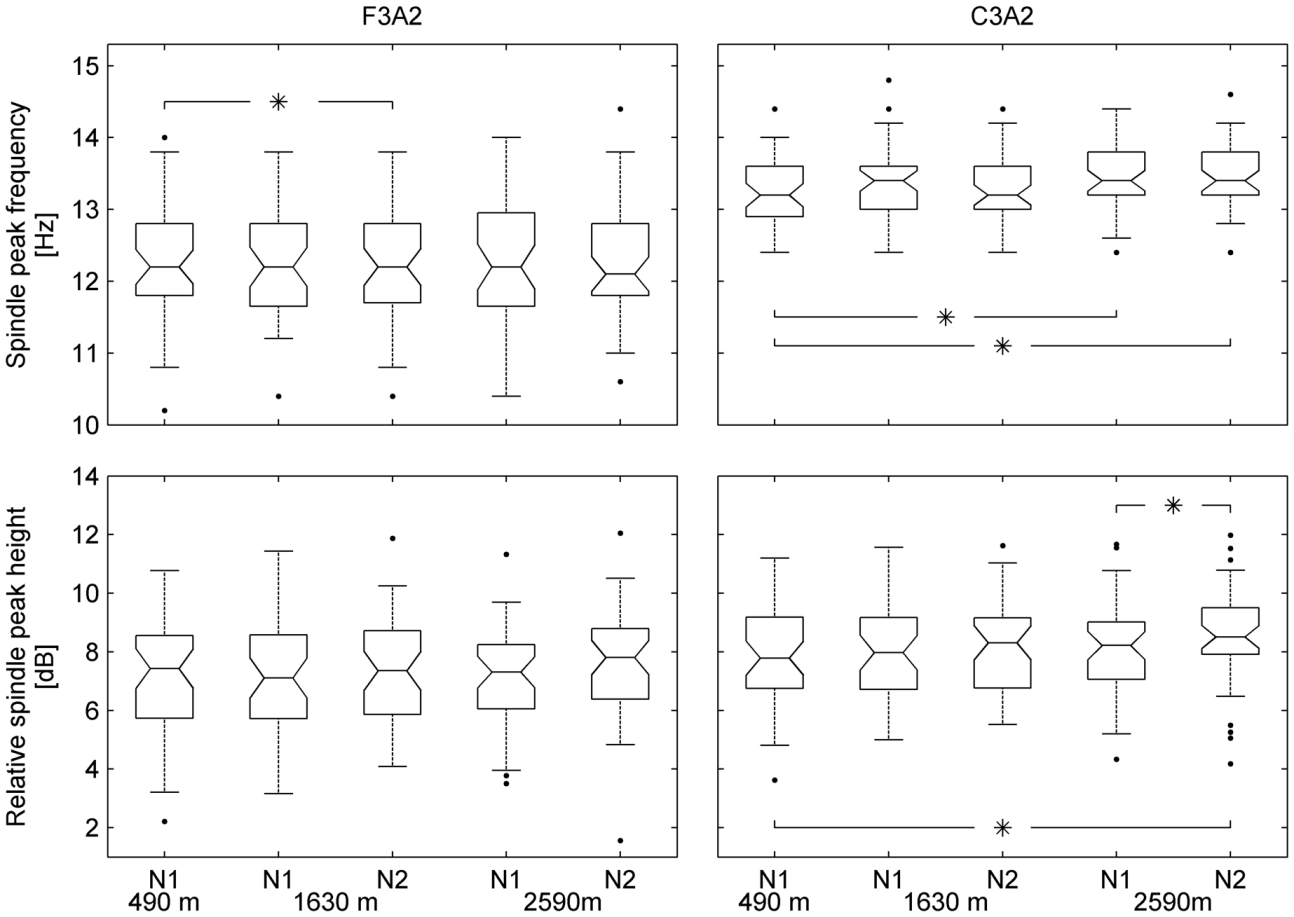
Sleep spindle characteristics. Spindle peak frequency: Average frequency of spindle peak in non-REM sleep EEG spectra. Relative spindle peak height: Peak height minus background activity at location of the peak. Data were derived from visually identified spindle peaks (n = 44; see Gottselig et al. [20]). The box plots show the lower quartile, median and upper quartile values. Whiskers include the adjacent values in the data up to values within one time the interquartile range. Outliers, displayed with ‘.’ are values beyond the ends of the whiskers. The notch indicates the 95% confidence interval of the median. *p<0.05: significant difference between baseline and altitude and between the first and second night (post-hoc paired t-test, whenever factor *Altitude* and/or *Night* were significant in the mixed model ANOVA with factors *Altitude*, *Night* and *Order*). N1, N2: night 1 and 2 at higher altitude.

We analyzed altitude dependent differences in sleep architecture (Table 1; Methods S1 and Table S1), spectral power (Figure 1 and 2; Table 2) and spindle variables (Figure 3) separately using SAS software (SAS 9.1.3; SAS Institute, Cary, NC, USA). *Post hoc* paired t-tests were performed if a mixed model ANOVA revealed significant effects.

**Table 2:** Changes in spectral variables at moderate altitude.

			1630 m N1	1630 m N2	2590 m N1	2590 m N2	p^A^
**SWA (change in %)**	F3A2	LH	−2.7 (5.4)	2.2 (4.5)	−14.1 (3.4)*	−10.5 (3.8)*	*<0.001*
		HL	−6.3 (3.2)*	−3.8 (4.2)	−18.0 (3.1)*	−16.7 (3.1)*	*<0.01*
	C3A2	LH	−1.8 (5.3)	2.9 (4.0)	−13.8 (2.7)*	−14.1 (3.7)*	*<0.0001*
		HL	−5.6 (4.6)	−2.5 (5.0)	−15.6 (3.3)*	−13.4 (2.7)*	*<0.05*
**Theta activity (change in %)**	F3A2	LH	−1.6 (3.6)	0.5 (3.3)	−8.9 (2.6)*	−5.8 (3.2)*	*<0.01*
		HL	−3.2 (3.1)	0.6 (3.7)	−14.8 (3.3)*	−14.3 (3.3)*	*<0.001*
	C3A2	LH	−0.2 (3.6)	3.1 (3.3)	−9.5 (2.4)*	−7.1 (4.1)*	*0.0001*
		HL	1.3 (2.8)	7.1 (3.5)	−12.0 (2.7)*	−11.8 (2.3)*	*<0.0001*
**Relative spindle peak height (change in %)**	F3A2	LH	4.5 (3.4)	11.2 (6.5)	8.5 (3.8)	8.5 (3.9)	*ns*
		HL	1.8 (3.8)	2.5 (3.6)	2.9 (4.0)	5.8 (3.9)	*ns*
	C3A2	LH	1.3 (3.4)	3.5 (3.0)	8.2 (3.2)*	12.2 (3.1)*	*<0.001*
		HL	6.8 (3.4)	6.9 (3.7)	3.2 (2.8)	7.1 (3.7)	*ns*

Variables are provided as change in percent of baseline. To take the order-effect of altitude exposure into account, the statistics were computed for the two groups LH (ascending from lower to higher altitude, *n* = 23) and HL (descending from higher to lower altitude, *n* = 21) separately. Data are provided as means (SEM). *Post hoc* t-tests were performed if factor *Condition* (p^A^; ns: not significant) of the mixed model ANOVA with factors *Condition* and *Order* of altitude exposure was significant.

* p<0.05 (paired t-test performed with log-transformed values) altitude compared to baseline. No differences were observed between the groups HL and LH (unpaired t-test).

F3A2: Frontal derivation; C3A2: Central derivation; SWA: Slow-wave activity (0.8–4.6 Hz); Theta activity: Power in the 4.6–8 Hz range; Relative spindle peak height: Height of the spindle peak minus background activity at position of the peak; N1, N2: night 1 and 2 at higher altitude.

### Statistical Analysis: Sleep EEG spectra and sleep variables

To examine the impact of altitude on the sleep EEG spectra, a linear mixed model ANOVA with factors *Condition* (490 m N1, 1630 m N1, 1630 m N2, 2590 m N1 and 2590 m N2; within subject), *Order* (group1, group2, group3, group4; between subjects) and their interaction was performed for each frequency bin separately for non-REM (Figure 1) and REM sleep at both derivations. Single bins may reach significance by chance, but would not be clustered in a band. Thus, only if ≥6 consecutive frequency bins (a range of 1.2 Hz) showed a significant change we would consider it relevant for our interpretation. To investigate changes in sleep structure (e.g., sleep latency, sleep efficiency and stages) and sleep cycles (e.g., total number of cycles, length of non-REM and REM episodes) at altitude, we used a mixed model ANOVA with factors *Altitude* (490 m, 1630 m and 2590 m), *Night* (N1 and N2), *Order* (group1, group2, group3, group4) and *Derivation* (F3A2 and C3A2). Furthermore, we used the same statistical model to test altitude dependent changes in two oscillatory bands which are homeostatically regulated – Slow wave activity (SWA: 0.8–4.6 Hz) and theta activity (4.6–8 Hz).

### Statistical analysis: Sleep spindles

Individual peaks in the spindle frequency range of 10 to 15 Hz were determined based on visual inspection of the all-night non-REM sleep power density spectra. If more than one peak was present, the peak with the higher frequency (fast spindles) was selected. Spindle peak height was calculated as height of the individual peak minus background activity (see Gottselig et al. [20] for details). Similar to SWA and theta activity we used a mixed model ANOVA with factors *Altitude*, *Night* and *Order* to examine spindle activity variables (e.g., frequency (Hz) and peak height).

### Statistical analysis: Order of altitude exposure

To evaluate whether the order of altitude exposure had an effect on sleep EEG characteristics, breathing variables and oxygen saturation, we divided subjects into two groups based on the order of exposure to altitude (excluding order of the baseline at Zurich; see Methods S1 and Figure S1). The group HL consisted of subjects descending from higher to lower altitude while in the group LH subjects were ascending from lower to higher altitude.

### Statistical analysis: Correlations between spectral power and breathing variables

Furthermore, we examined whether spectral power was related to changes in breathing and oxygen saturation (central AHI, ODI and SpO_2_). Based on an exploratory analysis (see Methods S1), mean power of three specific frequency bands (SWA, fast theta: 6.4–8 Hz and fast sigma: 13–14.4 Hz) was correlated (Spearman rank correlation) with respiratory measures and order effects were controlled for (level of significance α<0.05; Table 3).

**Table 3:** Correlations between spectral power and breathing variables.

		1630 m N1	1630 m N2	2590 m N1	2590 m N2
**Slow-wave activity (0.8–4.6 Hz)**					
**Central AHI (events/h)**	F3A2	ns	−0.52*	−0.33*	−0.31*
	C3A2	ns	−0.51*	−0.34*	−0.35*
**ODI (events/h)**	F3A2	ns	ns	−0.34*	ns
	C3A2	ns	ns	−0.40*	ns
**Fast theta activity (6.4–8 Hz)**					
**SpO**_2_ **(%)**	F3A2	ns	ns	0.38*	ns
	C3A2	ns	ns	0.41*	0.32*
**Fast sigma activity (13–14.4 Hz)**					
**SpO**_2_ **(%)**	F3A2	ns	ns	0.35*	ns
	C3A2	ns	ns	0.38*	ns

Spearman partial correlation coefficients (*p<0.05; ns, no significant correlation) corrected for *Order* of altitude exposure were determined to analyze the relationship of spectral power in specific frequency bands of the non-REM sleep EEG (slow-wave activity, fast theta activity, fast and slow sigma activity; frequency bands were determined in an explorative analysis (see Methods S1)) and altitude-related breathing variables (n = 44).

Mean oxygen saturation (SpO_2_), central apnea/hypopnea index (AHI) and oxygen desaturation index (ODI) was averaged for non-REM sleep. The number of arousals was calculated over total sleep time. F3A2: Frontal derivation; C3A2: Central derivation; N1, N2: night 1 and 2 at higher altitude.

## Results

### Sleep architecture

Exposure to moderate altitude had minor, but significant, effects on sleep architecture. In particular, the percentage of SWS (% of total sleep time) at 2590 m was 3.5% (N1) and 2.3% (N2) less than at 490 m, what corresponds to a reduction of 14.1% (N1) and 9.2% (N2) compared to baseline (Table S1; for data on all 51 subjects see Latshang et al. [12]). In the second night at both altitudes REM sleep percentage was increased (1630 m = 6% and 2590 m = 2.9%) compared to 490 m and sleep latency was reduced between 4 and 5 min (N1 and N2 at 2590 m; N1 at 1630 m). Total sleep time (N2 at 1630 m = 8 min and N2 at 2590 m = 6 min) and sleep efficiency (1630 m = 2.1% and 2590 m = 1.1%) were slightly increased on the second night at both altitudes compared to 490 m. The two groups LH (ascending from lower to higher altitude) and HL (descending from higher to lower altitude) differed only in the duration of REM sleep and stage 1 at the lower altitude (Table S1). Sleep cycle analysis confirmed that changes in the duration of non-REM and REM episodes at moderate altitude were marginal (Table 1). The number of cycles was slightly increased at 2590 m compared to baseline and the duration of the non-REM sleep episode and SWS duration were primarily affected in the second cycle.

### Spectral analysis: non-REM sleep EEG

Relative non-REM sleep EEG power density spectra (altitude/baseline) of frontal and central derivation are depicted in Figure 1. Decline in power was observed with increasing altitude for both derivations in the frequency range of 0.8–11.6 Hz, while power between frequencies 13.6–15.4 Hz increased (mixed model ANOVA with factors *Condition* and *Order*). Exposure to altitudes of 1630 m and 2590 m decreased EEG spectral power in the lower frequency range (0.8–11.4 Hz (F3A2) and 0.8–12.8 Hz (C3A2); Figure 1, *post-hoc* paired t-tests) compared to baseline. Thus, we further examined the effect of altitude on SWA (0.8–4.6 Hz) and theta activity (4.6–8 Hz; Figure 2).

SWA in non-REM sleep was reduced in an altitude-dependent manner, by 4.4% (p<0.05) in the first night at 1630 m in derivation F3A2 only and 16% (N1) and 13% (N2) in F3A2 as well as 15% (N1) and 14% (N2) in C3A2 (p<0.0001) at 2590 m compared to 490 m. As demonstrated previously [21], [22], SWA showed a predominance in the frontal derivation independent of altitude condition (mixed model ANOVA factor *Derivation*: p<0.0001).

Theta activity was reduced by 12% (N1) and 10% (N2) in F3A2 and by 11% (N1) and 9% (N2) in C3A2 (p<0.0001) at the highest altitude only.

### Spectral analysis: REM sleep EEG

SWA and theta activity were also reduced during REM sleep in both nights at 2590 m (p<0.0001; Figure 2). SWA was reduced by 9% (N1) and 11% (N2) in C3A2 only (p<0.01) and theta activity by 13% (N1) and 10% (N2) in F3A2 and 14% (N1) and 13% (N2) in C3A2 compared to 490 m.

### Sleep spindle activity

Although the mixed model ANOVA revealed an altitude effect in the frequency range of sleep spindles, *post-hoc* analysis did not show altitude related changes (Figure 1). The shape of the relative spectrum however, indicates that peaks in the spindle range may have been shifted. We therefore determined individual spindle peaks (see Methods). Figure 3 depicts the altitude-dependent changes in sleep spindle characteristics. Indeed, the spindle peak frequency in the central derivation was shifted to a slightly higher value at 2590 m (13.5 Hz) compared to baseline (13.3 Hz) on both nights.

Furthermore, on the second night at 2590 m, we observed an increase in relative spindle peak height compared to the baseline and the first night at 2590 m (Figure 3). In addition, we found a negative correlation between spindle peak height and SWA at 2590 m (2590 m = −0.35 (N1) and −0.33 (N2), p<0.05, Spearman rank correlation), but not at 1630 m where the sleep EEG was only slightly affected or at baseline.

### Spectral analysis in the two groups (LH and HL)

Detailed information on the effect of the order of altitude exposure on non-REM SWA, theta activity and spindle peak height was obtained by analyzing the subjects in two groups (LH, HL; Table 2). SWA and theta activity at 2590 m were reduced in both groups while relative spindle peak height at 2590 m was only increased in the LH group (C3A2; Table 2). There were no other differences between the groups HL and LH.

### Associations between spectral power and breathing variables/oxygen saturation

We found moderate associations between spectral power in different frequency bands and the central AHI, ODI and SpO_2_ at altitude (Table 3). Frequency bands for further analysis were identified in an exploratory analysis and correlation coefficients were computed (see Methods and Methods S1). SWA was negatively correlated with the number of central apneas and the resulting oxygen desaturation events, but not with SpO_2_. Furthermore, fast theta activity (6.4–8 Hz) and fast sigma activity (13–14.4 Hz; representing the power of fast spindles), were positively correlated with mean oxygen saturation during non-REM sleep. However, no associations with the central AHI and ODI were observed for theta and sigma activity.

### Acclimatization

The altitude-related decrease in SpO_2_ and increase in central AHI and ODI are depicted in Table S2. As shown by Latshang et al. [12] in 51 subjects, SpO_2_ at 2590 m was increased and central AHI at 1630 m and 2590 m was reduced on the second compared to the first night at altitude, indicating acclimatization to altitude.

In contrast to SpO_2_ and central AHI, SWA and theta activity in non-REM and REM sleep did not show signs of acclimatization, since no changes were observed between the first and second night at both altitudes (mixed model ANOVA factor *Night*: ns).

## Discussion

This is the first detailed report on spectral changes occurring in the non-REM and REM sleep EEG at moderate altitude investigated in a large healthy cohort and conducted in the field. Consistent with the changes in sleep architecture, we observed a considerable decline in SWA in an altitude-dependent manner together with an increase in spindle power/frequency at moderate altitude. With regards to breathing variables, central AHI and ODI were increased at moderate altitude, SpO_2_ was decreased, and EEG power in distinct frequency bands was associated with changes to respiratory signals. SWA was negatively correlated with central AHI and ODI, while fast theta and spindle activity correlated positively with SpO_2_. These findings provide evidence that hypoxia and breathing disturbances affect the sleep EEG even at moderate altitudes frequently visited during recreational mountain travels.

### Decrease in SWA and theta activity and increase in spindle activity

We investigated sleep at three different altitudes to assess the impact of moderate altitude on sleep EEG variables and their relationship to oxygen saturation and breathing disturbances. Exposure to hypobaric hypoxia decreased SWA in an altitude-dependent manner. SWA, a marker for sleep intensity and sleep homeostasis, increasing with time awake and dissipating over the course of the night depends on previous sleep-wake history [23]–[27]. A suppression of SWA may signal reduced dissipation of sleep pressure during a stay at moderate altitude and could lead to increased sleep propensity during the following days and in subsequent nights. Support for this hypothesis comes from the slight increases in total sleep time and sleep efficiency observed in the second night at both altitudes as well as the reduction in sleep latency (Table S1). The limitation of the sleep opportunity to 7 hours may also contribute to these effects on total sleep time and sleep efficiency. Thus, SWA might have been reduced even further without such confounds. On the other hand, the increase in SpO_2_ due to acclimatization on the second day at both altitudes may be responsible for the slight increases in total sleep time and sleep efficiency and the shorter sleep latency (Tables S1 and S2). Nussbaumer-Ochsner et al. [6] investigated sleep in the first and third night during a 3-day sojourn to 4550 m and showed that SWS was reduced at altitude, but increased from the first to the third night. They reported a positive effect of oxygen saturation on slow wave sleep at altitude after controlling for the AHI. We therefore suggest a similar effect on other sleep measures such as total sleep time and sleep latency. A reduction in SWA by 15% at 2590 m could result in considerable increases in sleep propensity. However, subjective sleepiness as assessed by the Karolinska sleepiness scale did not differ [12]. Also psychomotor and cognitive performance was not affected at this moderate altitude [12]. This indicates either that the applied tests were not sensitive enough or our observed altitude related EEG changes are too small to be of functional relevance.

Our results of a decrease in SWA are in line with Hoshikawa et al. [8] who found a 21% decline in SWA at a simulated altitude of 2000 m. This study was, however, non-randomized and performed in a small sample (8 male athletes) under simulated conditions rather than in the field. Athletes performed their training during the day at 22 m altitude, and were only exposed to normobaric hypoxia in a hypoxic room at a simulated altitude of 2000 m for 8 hours during the night. Therefore, the generalizability of these results to real world conditions is limited.

This is the first study to examine the impact of moderate altitude on REM sleep EEG power spectra. Similar to the changes observed in non-REM sleep, we found a reduction in SWA and theta activity at 2590 m in REM sleep. Therefore, we propose that hypoxia induced changes may have a common mechanism (see hypothesis below).

In addition, to the decline in SWA and theta activity we found an increase in spindle activity. Sleep spindles are generated in the thalamus resulting from the interaction of thalamic reticular inhibitory neurons and excitatory thalamo-cortical neurons that project to the cortex [28]. Also slow-waves are generated by thalamo-cortical loops, however, at a more hyperpolarized membrane potential than spindles [28]. We hypothesize that a reduced hyperpolarization of thalamic neurons for example due to sympathetic activation at altitude might therefore favor spindles and in turn suppress slow-waves. This could account for both, the decrease in SWA and the increase in spindle activity at moderate altitude. This hypothesis is supported by the negative correlation between spindle peak height and SWA at 2590 m, where we observed a distinct decrease in SWA and an increase in spindle activity, but not at lower altitude or baseline.

### Correlation between spectral power and breathing variables/oxygen saturation

We found a negative correlation between SWA and the central AHI and ODI at 2590 m suggesting a relationship between sleep intensity and respiratory variables. The decrease in SWA may therefore partly be due to disruption of sleep through breathing disturbances. Our findings are in agreement with results from studies in patients with obstructive sleep apnea syndrome (OSAS), where AHI and SWS are negatively correlated [29] and SWS, SWA and theta activity are reduced compared to healthy controls [30], [31]. Frequent central apneas might therefore be one reason for a reduction in SWA at moderate altitude.

The relationship between sleep quality at altitude and breathing variables has two aspects. The first aspect is the relationship between the increased number of central apneas, as measured by AHI and periodic breathing, and sleep quality. Studies examining this relationship have found weak associations between breathing disturbances and arousals, although both worsen with increasing altitude [6], [32], [33]. The current study, which was conducted at a lower altitude than previous studies, is the first to examine the association between AHI and sleep EEG spectral characteristics. We found a correlation between AHI and SWA, indicating that perhaps increased AHI results in subtle sleep disruption.

The second aspect is the relationship between oxygen saturation and sleep quality. Previous studies reported a positive relationship between these variables [5], [6]. Using spectral analysis we found a correlation between fast theta and sigma activity and oxygen saturation. Thus, different frequency bands are correlated with changes in oxygen saturation and breathing parameters at moderate altitude, suggesting that spectral analysis may be uniquely able to differentiate changes due to these parameters.

### Acclimatization to altitude

Our unique study design allowed for the examination of altitude-dependent effects and changes over several days at altitude. It is known that oxygen saturation increases in the course of acclimatization to altitude [34]. Previous studies regarding the progression of AHI during a prolonged stay at altitude, however, suggest that central apneas and periodic breathing does not normalize [6]. Bloch et al. [35] examining 34 mountaineers on a high altitude expedition to Mount Muztagh Ata (7546 m) observed that the apnea/hypopnea index continued to increase during repeated recordings at 4497 m. In contrast, we observed signs of acclimatization in oxygen saturation and respiratory variables (Table S2; Latshang et al. [12]). Subjects, which spent their first night at 1630 m showed higher AHI and lower SpO_2_ compared to subjects sleeping at this altitude after two nights at higher altitude (2590 m).

Little is known about the development of altitude-dependent changes in sleep over the course of acclimatization, but changes in sleep stages and the increased number of arousals are thought to return towards sea level values after several days at altitude [3], [9], [36]. In a field study at 4559 m [6], SWS increased from 6% in the first night to 11% in the third one. We did not find any significant altitude-order effects in the spectral data, although the reduction in SWA and theta activity was more pronounced at 2590 m in subjects ascending first to the highest altitude (HL group) and SWS was only reduced in this group (Table S1).

### Limitations

One limitation of the study was that our subjects did not have an adaptation night. We tried to minimize first night effects by the randomization of the subjects into four groups and by only including subjects that had a sleep efficiency of ≥80% on the first experimental night. Dividing the subjects into two groups (LH vs. HL) allowed us to examine order effects in altitude exposure, revealing important findings. The power of this analysis, however, was reduced due to the smaller sample size in the subgroups. Additionally, participants were only allowed to sleep 7 hours, which may have resulted in a higher sleep propensity during subsequent nights. A further limitation is that men only were investigated.

### Conclusion

Tourism to moderate altitude for recreation is common worldwide. Such trips are often accompanied by daily sports activities (e.g. skiing, climbing, mountainbiking) requiring fine motor control and cognitive engagement. Sleep studies at normobaric conditions have shown detrimental effects of sleep disruption on motor and cognitive tasks [37]. Spectral analysis is particularly well-suited to detect such minor changes in the sleep EEG. The current study shows that already at moderate altitudes considerable alterations in the sleep EEG are evident and correlations exist between breathing parameters/oxygen saturation and specific frequency bands.

## Supporting Information

Methods S1 **Supplemental Methods.**
(DOCX)

Figure S1 **Randomization into four groups.** The 44 subjects were randomized into four groups with different order of altitude exposure. First, the baseline night in Zurich was either scheduled before or after the four consecutive nights at moderate altitude. The interval between baseline and sessions at altitude was 4 to 10 weeks. Second, the order of the stay in Davos Wolfgang and on the Jakobshorn (two nights each) was randomized.
(TIF)

Table S1 **Sleep variables derived from visual scoring.** Data are provided as mean values with standard deviation in parenthesis. Listed are the values averaged over all 44 subjects (TM) and for the two subgroups LH (ascending from lower to higher altitude, n = 23) and HL (descending from higher to lower altitude, n = 21). The difference between the two groups was evaluated by comparing the differences between baseline and altitude values. Post hoc test were performed if factor Condition (pA) of the mixed model ANOVA with factors Condition and Order of altitude exposure was significant. *p<0.05 (Wilcoxon signed rank test) altitude compared to baseline. ‡p<0.05 (Mann-Whitney U test) Comparison of the difference baseline-altitude between the group HL and the group LH. Sleep efficiency is total sleep time as a percentage of time in bed. Sleep stages are reported as percentage of total sleep time. Sleep latency was measured as interval from lights off to the first occurrence of stage 2 sleep. Slow wave sleep is the sum of stage 3 and 4. Time in bed was 7 h.
(DOCX)

Table S2 **Breathing variables and oxygen saturation.** Data are provided as medians with median deviation in parenthesis. Listed are the medians over all 44 subjects (TM) and for the two subgroups LH (ascending from lower to higher altitude, n = 23) and HL (descending from higher to lower altitude, n = 21). The difference between the two groups in the central apnea/hypopnea index (AHI) and oxygen desaturation index (ODI) was evaluated by comparing the differences between altitude and baseline values of the two groups. For oxygen saturation (SpO2) the absolute values were compared. *p<0.05, +p<0.1 (Wilcoxon signed rank test) altitude compared to baseline. ‡p<0.05 (Mann-Whitney U test) Comparison of the difference baseline-altitude between the groups HL and LH. Oxygen saturation, central apnea/hypopnea index and oxygen desaturation index were averaged over total non-REM sleep.
(DOCX)
