# Supplementary material for: Quantitative Changes in the Sleep EEG at Moderate Altitude (1630 m and 2590 m)
Source: PLoS One. 2013 Oct 22;8(10):e76945. doi: 10.1371/journal.pone.0076945 (PMC3805553; doi:10.1371/journal.pone.0076945)
Supplement: Methods S1 — Supplemental Methods. (DOCX) [file pone.0076945.s001.docx]

**Methods S1**

*Randomization*

Subjects were randomized into four groups with different order of altitude exposure (Figure S1).

For more specific analyses subjects were divided into the two groups LH (ascending from lower to higher altitude; group 1 and 3) and HL (descending from higher to lower altitude; group 2 and 4), neglecting the time point of the baseline.

*Statistics sleep stage analysis*

Because different persons were involved in the sleep stage scoring, all scored nights were reviewed by a single person to assure concordance of the scoring within and between subjects.

A linear mixed model ANOVA (SAS 9.1.3; SAS Institute, Cary, NC, USA) with factors *Condition* (490m N1, 1630m N1, 1630m N2, 2590m N1 and 2590 m N2; within subject) and *Order* (group1, group2, group3, group4; between subjects) and their interaction was performed. *P*ost hoc Wilcoxon rank sum tests were performed for sleep stage variables if factor *Condition* or the interaction *Condition* x *Order* reached significance (p < 0.05).

To evaluate order effects of altitude exposure, the two sub-groups (HL: descending from higher to lower altitude [groups 2 and 4] and LH: ascending from lower to higher altitude [groups 1 and 3]) were analyzed separately. Again, a linear mixed model ANOVA including the factors *Condition* and *Order* (baseline before or after altitude exposure) was performed.

Sleep variables of the HL and LH groups were compared by non-parametric Mann-Whitney U-tests (Table S1).

*Statistics spectral analysis: Comparison of the two subgroups (LH and HL)*

To evaluate whether the order of altitude exposure had an effect, the two groups (HL: descending from higher to lower altitude [groups 2 and 4] and LH: ascending from lower to higher altitude [groups 1 and 3]; Figure S1) were analyzed separately by linear mixed model ANOVA including the factors *Condition* and *Order* (see above). *Post hoc* paired t-tests were performed as described above. In addition, altitude effects on sleep EEG characteristics of the two groups were compared using relative values (value at each altitude [e.g. 1630 m N1] was divided by the baseline value, Table 2).

*Breathing variables*

A corresponding analysis as above was performed for breathing variables and oxygen saturation (Table S2).

*Partial correlation between spectral power and respiratory variables*

We explored associations between different physiological variables (central AHI, ODI and SpO2) and the spectral power in each frequency bin by Spearman rank correlations. Single bins may reach a significant correlation by change, but would not be clustered in a band. Thus, at least 6 consecutive frequency bins (0.2 Hz) had to reach significance to be considered relevant for further analysis and interpretation. Based on this explorative analysis power of three specific frequency bands (SWA, fast theta: 6.4-8 Hz and fast sigma: 13-14.4 Hz) was calculated and used for partial correlations with central AHI, ODI and SpO2.
