# Supplementary figures and images for: Quantitative Changes in the Sleep EEG at Moderate Altitude (1630 m and 2590 m)
Source: PLoS One. 2013 Oct 22;8(10):e76945. doi: 10.1371/journal.pone.0076945 (PMC3805553; doi:10.1371/journal.pone.0076945)

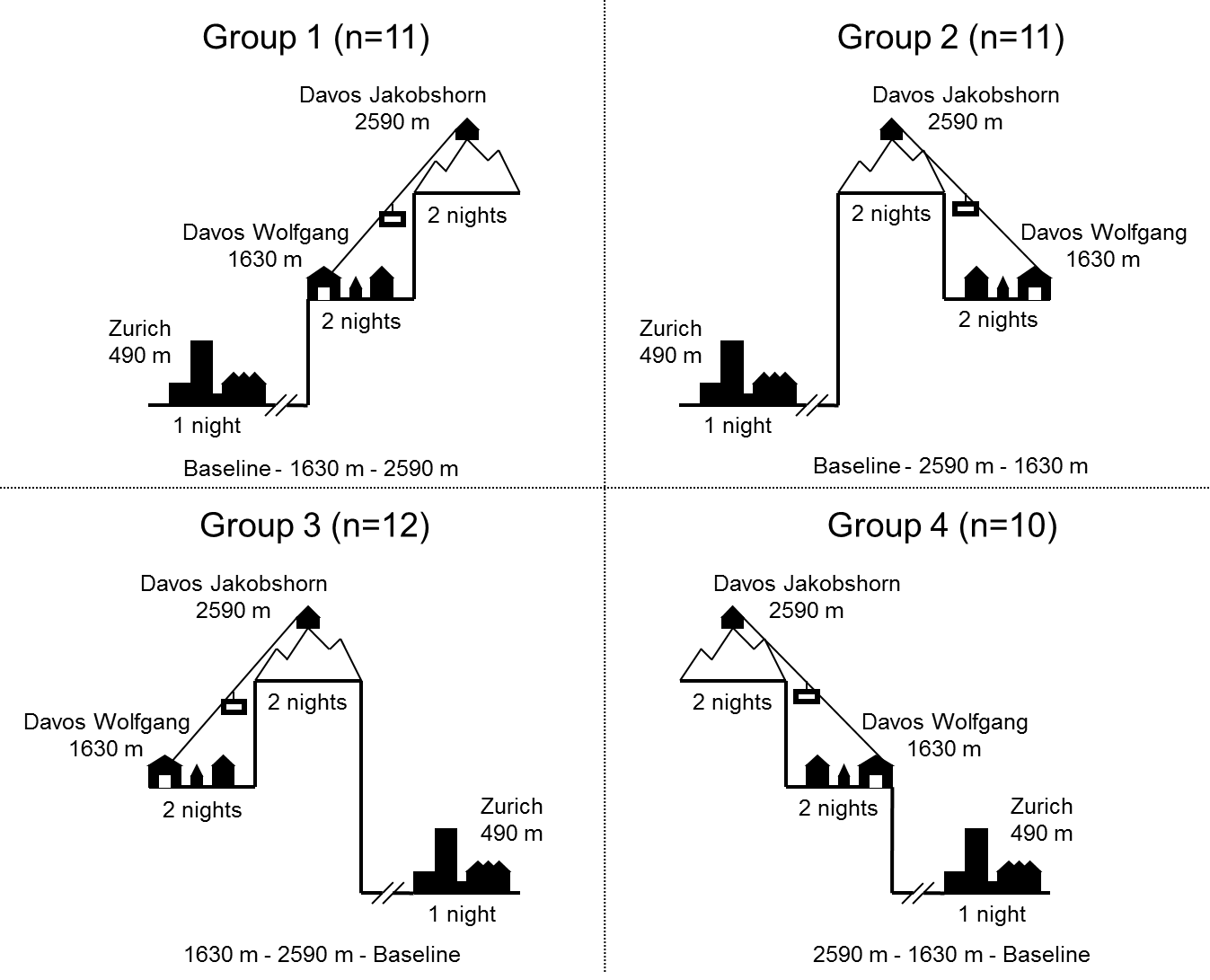

Supplement: Figure S1 — Randomization into four groups. The 44 subjects were randomized into four groups with different order of altitude exposure. First, the baseline night in Zurich was either scheduled before or after the four consecutive nights at moderate altitude. The interval between baseline and sessions at altitude was 4 to 10 weeks. Second, the order of the stay in Davos Wolfgang and on the Jakobshorn (two nights each) was randomized. (TIF) [file pone.0076945.s002.tif]
