# Supplementary material for: Quantitative Changes in the Sleep EEG at Moderate Altitude (1630 m and 2590 m)
Source: PLoS One. 2013 Oct 22;8(10):e76945. doi: 10.1371/journal.pone.0076945 (PMC3805553; doi:10.1371/journal.pone.0076945)
Supplement: Table S1 — Sleep variables derived from visual scoring. Data are provided as mean values with standard deviation in parenthesis. Listed are the values averaged over all 44 subjects (TM) and for the two subgroups LH (ascending from lower to higher altitude, n = 23) and HL (descending from higher to lower altitude, n = 21). The difference between the two groups was evaluated by comparing the differences between baseline and altitude values. Post hoc test were performed if factor Condition (pA) of the mixed model ANOVA with factors Condition and Order of altitude exposure was significant. *p<0.05 (Wilcoxon signed rank test) altitude compared to baseline. ‡p<0.05 (Mann-Whitney U test) Comparison of the difference baseline-altitude between the group HL and the group LH. Sleep efficiency is total sleep time as a percentage of time in bed. Sleep stages are reported as percentage of total sleep time. Sleep latency was measured as interval from lights off to the first occurrence of stage 2 sleep. Slow wave sleep is the sum of stage 3 and 4. Time in bed was 7 h. (DOCX) [file pone.0076945.s003.docx]

**Table S1: Sleep variables derived from visual scoring**

|  |  | | **490 m** | | **1630 m N1** | **1630 m N2** | **2590 m N1** | **2590 m N2** | **p^A^** |
| --- | --- | --- | --- | --- | --- | --- | --- | --- | --- |
| **Total sleep time (min)** | | **TM** | | **393.1 (20.2)** | **388.4 (34.0)** | **401.4 (14.5)*** | **396.0 (15.0)** | **399.5 (14.0)*** | **<0.01** |
|  |  | LH | | 391.9 (17.4) | 384.9 (26.8) | 398.3 (17.3) | 398.9 (13.6)* | 397.6 (14.5) | <0.01 |
|  |  | HL | | 394.4 (23.2) | 392.2 (40.8) | 404.4 (10.7) | 392.9 (40.8) | 401.5 (13.6) | ns |
| **Sleep efficiency (%)** | | **TM** | | **0.94 (0.04)** | **0.93 (0.08)** | **0.96 (0.03)*** | **0.94 (0.04)** | **0.95 (0.03)*** | **<0.01** |
|  |  | LH | | 0.93 (0.04) | 0.92 (0.06) | 0.95 (0.04) | 0.95 (0.03)* | 0.95 (0.03) | <0.01 |
|  |  | HL | | 0.94 (0.04) | 0.93 (0.10) | 0.96 (0.03) | 0.94 (0.04) | 0.96 (0.03) | ns |
| **Sleep latency (min)** | | **TM** | | **13.2 (7.5)** | **11.5 (8.5)** | **9.3 (4.2)*** | **9.1 (5.0)*** | **8.3 (4.7)*** | **<0.0001** |
|  |  | LH | | 12.4 (5.7) | 11.9 (8.7) | 9.6 (3.7)* | 8.3 (3.7)* | 8.0 (3.1)* | <0.001 |
|  |  | HL | | 14.1 (9.2) | 11.0 (8.5) | 9.0 (4.7)* | 10.0 (6.1) | 8.6 (6.1)* | <0.05 |
| **Non-REM sleep (%)** | | **TM** | | **70.5 (5.8)** | **68.7 (5.0)** | **67.9 (6.6)** | **68.0 (7.0)*** | **67.6 (5.5)*** | **<0.05** |
|  |  | LH | | 70.2 (5.2) | 69.0 (5.9) | 68.8 (7.6) | 68.8 (6.2) | 66.9 (5.6) | ns |
|  |  | HL | | 70.7 (6.5) | 68.4 (3.9) | 67.0 (5.6) | 67.2 (7.8) | 68.5 (5.5) | 0.06 |
| **REM sleep (%)** | | **TM** | | **20.3 (5.7)** | **22.2 (5.5)** | **26.3 (5.7)*** | **22.4 (6.2)** | **23.2 (4.8)*** | **<0.0001** |
|  |  | LH | | 21.3 (5.1) | 20.6 (6.2)‡ | 25.7 (6.3)‡ | 23.1 (5.6) | 24.1 (4.8)* | <0.01 |
|  |  | HL | | 19.2 (6.2) | 24.0 (4.1)*‡ | 27.0 (5.0)*‡ | 21.7 (6.9) | 22.1 (4.6)* | <0.0001 |
| **Stage 1 (%)** | | **TM** | | **9.2 (4.9)** | **9.1 (4.7)** | **5.8 (2.6)*** | **9.6 (5.6)** | **9.2 (4.9)** | **<0.0001** |
|  |  | LH | | 8.4 (4.1) | 10.5 (5.5)‡ | 5.5 (2.5)* | 8.1 (3.8) | 9.0 (4.8) | <0.001 |
|  |  | HL | | 10.1 (5.7) | 7.6 (3.2)‡ | 6.0 (2.7)* | 11.2 (6.8) | 9.4 (5.2) | <0.001 |
| **Stage 2 (%)** | | **TM** | | **45.6 (8.5)** | **44.7 (7.3)** | **41.9 (8.4)*** | **46.7 (7.9)** | **45.1 (6.7)** | **<0.01** |
|  |  | LH | | 46.5 (9.0) | 45.1 (7.5) | 42.0 (8.4)* | 47.2 (8.0) | 43.7 (7.5) | <0.05 |
|  |  | HL | | 44.6 (7.9) | 44.3 (7.2) | 41.8 (8.7) | 46.2 (8.1) | 46.7 (5.5) | <0.05 |
| **Slow wave sleep (%)** | | **TM** | | **24.8 (7.0)** | **24.0 (5.7)** | **26.0 (5.9)** | **21.3 (5.7)*** | **22.5 (5.4)*** | **<0.0001** |
|  |  | LH | | 23.7 (6.2) | 23.9 (5.2) | 26.9 (5.0)* | 21.5 (5.5) | 23.2 (6.4) | <0.001 |
|  |  | HL | | 26.1 (7.6) | 24.1 (6.4) | 25.2 (6.6) | 21.0 (6.1)* | 21.8 (4.2)* | <0.0001 |
| **Waking after sleep onset (min)** | | **TM** | | **12.4 (12.6)** | **19.9 (28.9)** | **9.2 (12.8)** | **14.1 (14.4)** | **11.7 (12.4)** | **<0.05** |
|  |  | LH | | 15.0 (15.3) | 22.5 (22.3) | 11.8 (16.6) | 12.3 (14.4) | 14.0 (14.3) | <0.05 |
|  |  | HL | | 9.5 (8.3) | 17.2 (35.2) | 6.7 (7.4) | 16.2 (14.5) | 9.1 (9.7) | ns |
