# Supplementary material for: Quantitative Changes in the Sleep EEG at Moderate Altitude (1630 m and 2590 m)
Source: PLoS One. 2013 Oct 22;8(10):e76945. doi: 10.1371/journal.pone.0076945 (PMC3805553; doi:10.1371/journal.pone.0076945)
Supplement: Table S2 — Breathing variables and oxygen saturation. Data are provided as medians with median deviation in parenthesis. Listed are the medians over all 44 subjects (TM) and for the two subgroups LH (ascending from lower to higher altitude, n = 23) and HL (descending from higher to lower altitude, n = 21). The difference between the two groups in the central apnea/hypopnea index (AHI) and oxygen desaturation index (ODI) was evaluated by comparing the differences between altitude and baseline values of the two groups. For oxygen saturation (SpO2) the absolute values were compared. *p<0.05, +p<0.1 (Wilcoxon signed rank test) altitude compared to baseline. ‡p<0.05 (Mann-Whitney U test) Comparison of the difference baseline-altitude between the groups HL and LH. Oxygen saturation, central apnea/hypopnea index and oxygen desaturation index were averaged over total non-REM sleep. (DOCX) [file pone.0076945.s004.docx]

**Table S2: Breathing variables and oxygen saturation**

|  |  | **490 m** | **1630 m N1** | **1630 m N2** | **2590 m N1** | **2590 m N2** |
| --- | --- | --- | --- | --- | --- | --- |
| **SpO2 (%)** | **TM** | **96 (0.8)** | **94 (0.8)*** | **94 (0.7)*** | **90 (1.3)*** | **91 (1.1)*** |
|  | LH | 96 (0.9) | 93 (0.7)‡* | 94 (0.8)* | 90 (1.2)* | 91 (1.3)* |
|  | HL | 96 (0.6) | 94 (0.7)‡* | 94 (0.6)* | 90 (1.3)* | 91 (1.0)* |
| **Central AHI (events/h)** | **TM** | **2.0 (1.8)** | **4.8 (5.5)*** | **2.9 (3.1)*** | **9.1 (17.5)*** | **5.2 (9.5)*** |
|  | LH | 2.1 (2.3) | 7.4 (7.7)‡* | 3.6 (4.1)* | 8.9 (19.8)* | 5.0 (13.4)* |
|  | HL | 2.0 (1.5) | 2.8 (2.2)‡ | 2.9 (2.1)* | 9.5 (15.1)* | 6.3 (5.9)* |
| **ODI (events/h)** | **TM** | **0.2 (0.6)** | **1.6 (4.4)*** | **1.3 (2.8)*** | **5.6 (22.4)*** | **2.8 (12.3)*** |
|  | LH | 0.2 (0.7) | 2.0 (7.1)‡* | 1.5 (4.4)* | 8.5 (29.1)* | 2.8 (19.6)* |
|  | HL | 0.2 (0.4) | 1.2 (1.0)‡* | 1.2 (1.1)* | 4.2 (15.3)* | 2.8 (5.5)* |
| **Arousals (events/h)** | **TM** | **8.2 (2.1)** | **6.4 (2.1)*** | **6.7 (1.9)*** | **7.6 (2.6)** | **7.7 (3.0)** |
|  | LH | 8.3 (1.9) | 7.0 (2.8)‡ | 6.7 (2.4) | 7.2 (3.3) | 8.3 (3.5) |
|  | HL | 7.8 (2.3) | 5.9 (1.8)* | 6.9 (1.3) | 7.9 (1.8) | 7.0 (2.3) |
